# Calmodulin and Amyloid Beta as Coregulators of Critical Events during the Onset and Progression of Alzheimer’s Disease

**DOI:** 10.3390/ijms24021393

**Published:** 2023-01-11

**Authors:** Danton H. O’Day

**Affiliations:** 1 Cell and Systems Biology, University of Toronto, Toronto, ON M5S 3G5, Canada; danton.oday@utoronto.ca; 2 Department of Biology, University of Toronto Mississauga, Mississauga, ON L5L 1C6, Canada

**Keywords:** calmodulin binding proteins, amyloid beta receptors, Alzheimer’s disease, neurodegeneration, calcium regulation, amyloid pathway, NMDAR

## Abstract

Calmodulin (CaM) and a diversity of CaM-binding proteins (CaMBPs) are involved in the onset and progression of Alzheimer’s disease (AD). In the amyloidogenic pathway, AβPP1, BACE1 and PSEN-1 are all calcium-dependent CaMBPs as are the risk factor proteins BIN1 and TREM2. Ca^2+^/CaM-dependent protein kinase II (CaMKII) and calcineurin (CaN) are classic CaMBPs involved in memory and plasticity, two events impacted by AD. Coupled with these events is the production of amyloid beta monomers (Aβ) and oligomers (Aβo). The recent revelations that Aβ and Aβo each bind to both CaM and to a host of Aβ receptors that are also CaMBPs adds a new level of complexity to our understanding of the onset and progression of AD. Multiple Aβ receptors that are proven CaMBPs (e.g., NMDAR, PMCA) are involved in calcium homeostasis an early event in AD and other neurodegenerative diseases. Other CaMBPs that are Aβ receptors are AD risk factors while still others are involved in the amyloidogenic pathway. Aβ binding to receptors not only serves to control CaM’s ability to regulate critical proteins, but it is also implicated in Aβ turnover. The complexity of the Aβ/CaM/CaMBP interactions is analyzed using two events: Aβ generation and NMDAR function. The interactions between Aβ, CaM and CaMBPs reveals a new level of complexity to critical events associated with the onset and progression of AD and may help to explain the failure to develop successful therapeutic treatments for the disease.

## 1. Calmodulin Binding Proteins and Alzheimer’s Disease

**Figure 1:**
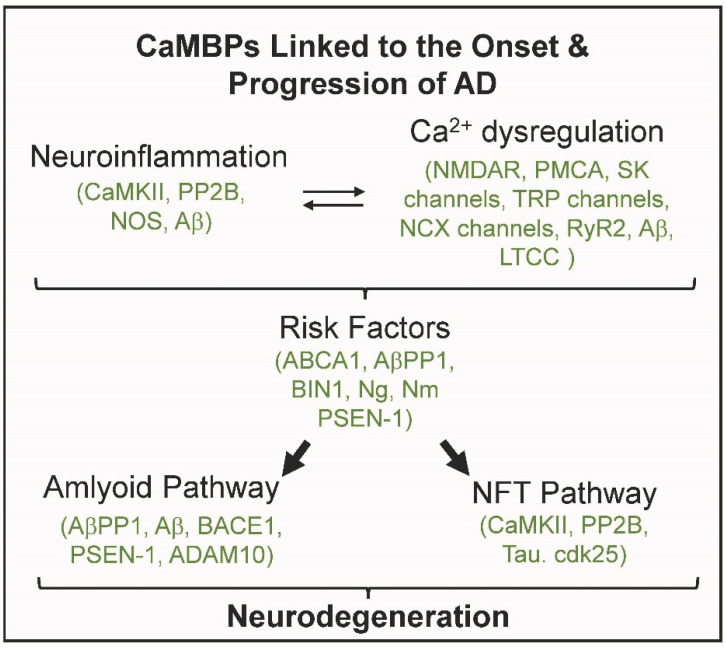
Experimentally validated calmodulin binding proteins (CaMBPs, Green) are involved in critical events in the onset and progression of Alzheimer’s disease.

While the initiating events of Alzheimer’s disease (AD) are controversial and still under analysis, risk factors, neuroinflammation and calcium dysregulation are widely accepted as precursor events to the resulting production of amyloid plaques, neurofibrillary tangles (NFTs) and neurodegeneration, the classic hallmarks of AD [1,2,3,4,5,6]. The importance of calcium dysregulation was recognized in the 1980s and continues to be a fundamental hypothesis for AD (Calcium Hypothesis) [2]. Calcium mainly works by binding to proteins of which calmodulin (CaM) is the primary brain calcium-binding protein [7]. The early and intimate relationship between CaM and AD has been well established by a multitude of researchers [8,9,10]. CaM binds to and regulates target CaM-binding proteins (CaMBPs) in most if not all AD. Over two dozen proteins linked to the onset and progression of AD are experimentally validated CaMBDs (Figure 1). These and other data continue to support the Calmodulin Hypothesis of AD [8]. 

The inter-connected events of calcium dysregulation and neuroinflammation occur early in AD and other neurodegenerative diseases and either activate risk factors or respond to them. Four proteins involved in AD neuroinflammation are experimentally proven CaMBPs (e.g., CaMKII, PP2B, NOS, Aβ) while at least eight mediate calcium dysregulation (e.g., NMDAR, PMCA, SK channels, TRP channels, NCX channels, RyR2, LTCC; Aβ; Figure 1; Table 1). Many AD risk factors have also been proven to be experimentally validated CaMBPs (e.g., ABCA1, AβPP, BIN1, Ng, Nm, PSEN-1; Figure 1). 

Calmodulin function is central to both the amyloid and NFT pathways of AD. CaM binds to AβPP, the precursor for Aβ production [12,29]. (Figure 1). BACE1, the first enzyme in the amyloid beta pathway, is a CaMBP as is PSEN-1, a component of the second enzyme γ-secretase [20,30]. The CaMBP ADAM10 is involved in redirecting AβPP1 processing along the non-amyloidogenic pathway [31]. As detailed below the product of BACE1 and γ-secretase is the peptide Aβ of which Aβ42, a peptide of 42 amino acids, appears to be the most toxic. CaM function extends to the NFT pathway where CaMKII and PP2B come into play again, as they do in other events, such as LTP, LTD and plasticity, that are not covered here [32,33]. Tau is a CaMBP that is phosphorylated (pTau) by many kinases including the CaMBP cdk25 prior to its oligomerization towards NFT formation [34,35]. In addition to experimentally validated CaMBPs, many putative CaMBPs involved in AD have been identified [8,9]. 

While the fine details of CaM’s regulatory involvement in the onset and progression of AD continue to be sorted out, it has recently been shown that Aβ binds directly to CaM and to multiple proteins involved in disease. Here we show that many AD-linked Aβ receptors are also CaMBPs adding new levels of complexity to our understanding of the onset and progression of AD. 

## 2. Aβ/CaMBP Receptors Involved in Alzheimer’s Disease

Over 100 potential Aβ/Aβo receptors have been identified in human brain extracts and their functions have been well reviewed [22,36,37,38]. Dozens of Aβ receptors are linked to neuroinflammation, calcium regulation and other critical events linked to normal brain function and neurodegenerative diseases including AD [22,37,38,39]. Of relevance here are those Aβ receptors that are also CaMBPs intimately linked to AD (Table 1). The interaction between CaM and those Aβ receptors can be divided into two primary groups: “Direct Regulation” (e.g., Aβ receptor is a CaMBP) or “Indirect Regulation” (e.g., Aβ receptor is not a CaMBP but is regulated by a CaMBP). Unless otherwise indicated the term Aβ will be used to indicate the different Aβ species and oligomers.

Aβ receptors that are experimentally validated CaMBPs that show Direct Regulation include: Aβ, AβPP1, mGluR, NMDAR, PMCA and PSEN1 (Table 1). Each of these CaMBPs bind to and are regulated by Aβ. They are discussed further below. Examples of Aβ receptors that show Indirect Regulation include α7nAChR, AMPAR and TREM2 (Table 1). These Aβ receptors are not CaMBPs but, as listed here, are regulated via the classic CaMBPs PP2B and CaMKII. Examples of direct and indirect regulation are detailed below revealing how they can also work together in the Combined Regulation involving Aβ receptors. 

While they will not be detailed here, several risk factors that are Aβ receptors that possess CaM-binding domains (i.e., are presumptive CaMBPs) also show direct regulation (Table 1). Present on the surface of microglia, TREM2 (triggering receptor expressed on myeloid cells 2) is a transmembrane-glycoprotein receptor that is a risk factor for AD that binds to Aβ [25,40]. (Table 1). CLU/ApoJ and PICALM are two other examples (Table 1). Three APOE isoforms (APOE 2-4) differentially bind to Aβ modulating its conversion to fibrils [22,41]. APOE has two potential CaMBDs with multiple binding motifs [9]. 

Thus, multiple Aβ receptors that are proven or presumptive CaMBPs are intimately involved in the onset and progression of AD. Since Aβ also binds to CaM, the regulatory implications become more complex. The two following examples will clarify this and provide more insight into the direct, indirect and combined regulation of Aβ receptors.

## 3. Aβ, CaM and Calcium Channels

The role of the glutamate receptors NMDAR and AMPAR in AD have been reviewed (Table 1) [42]. In addition to being both a CaMBP and Aβ receptor which opens them up for direct regulation, NMDAR are also indirectly regulated by CaMKII, thus setting them up for combined regulation. The intracellular C0 domain of the NMDAR NR1 subunit binds to apo-CaM [43]. It desensitizes the NMDAR until sufficient glutamate stimulation results in an influx of calcium ions that converts apo-CaM to Ca^2+^/CaM which, in turn, leads to the calcium-dependent inactivation (CDI) of the receptor and its release from the membrane [16]. CDI functions as an autoinhibitory mechanism to protect against unregulated calcium influx that could be cytotoxic. The resulting increase in local post-synaptic calcium ion levels also transforms cytoplasmic apo-CaM to Ca^2+^/CaM which, in turn, binds to and activates CaMKIIa. The kinase also binds to and likely potentiates NMDAR activity [44]. To add to this complex interaction, CaMKIIa phosphorylates AMPAR causing it to translocate to the membrane where it can interact with NMDAR. As part of this indirect regulation, the presence of Aβ prevents this translocation [45]. Evidence has also been presented that Aβ oligomers activate NMDARs containing GluN2B subunits [46,47]. While one group has presented evidence that this is a result of CaMKII activation by Aβ oligomers others have shown that Aβ oligomers inhibit CaMKII autophosphorylation [33]. Clearly the interplay between Aβ, Aβ receptors and CaM is potentially complex with multiple functions that have implications to AD. 

## 4. The Complex Interplay between CaM and Aβ in the Amyloid Pathway

**Figure 2:**
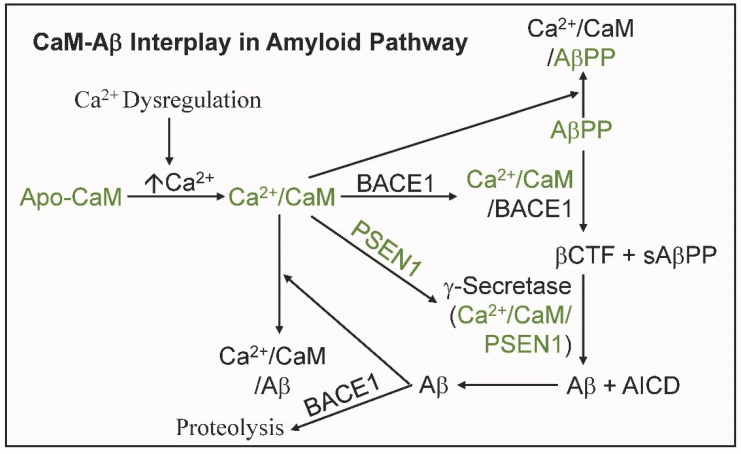
The interplay between calmodulin (CaM) and amyloid beta (Aβ) in the amyloid pathway. Calmodulin (CaM) and its binding proteins that bind to Aβ are shown in green.

The interplay between CaM and Aβ occurs at the start of the amyloidogenic pathway (Figure 2; Table 1). As covered above, several experimentally validated CaMBPs are involved in the initial generation of Aβ: AβPP, BACE1, PSEN1. CaM-binding to BACE1 increases enzyme activity 2.5-fold in vitro [30]. BACE1 activity is also increased in both early onset and late onset forms of AD and by PSEN1, mutations apparently through the resulting increased generation of Aβ that activates BACE1 gene transcription increasing the level of this primary enzyme in the amyloidogenic pathway [48]. Thus Aβ provides a positive feed-back loop in its own production [38]. Aβ42 levels are dependent not only on their production via the sequential degradation of AβPP by BACE1 and γ-secretase but also by their depletion as they oligomerize and form fibrils on their pathway to plaque formation. Since soluble Aβ oligomers are transient, they can re-release Aβ42 monomers [49]. To add to this, as a cysteine protease, BACE1 is also involved in Aβ degradation [50]. 

CaM-binding to AβPP regulates the non-amyloidogenic pathway while PSEN-1 binding to CaM has been shown to function in the regulation of intracellular calcium levels (Figure 2; Table 1) [12,20]. Once Aβ is produced it feeds back on its synthesis via its binding to both apo- and Ca^2+^/CaM, AβPP and PSEN1 [11,13,21]. The binding of Aβ to AβPP is a complex issue that has been reviewed but leaves the question of significance unanswered [5]. That is not the case for PSEN1, a catalytic subunit of γ-secretase. Aβ42 binds to transmembrane domain 1 (TMD1) of PSEN1, a region that modulates Aβ generation, with resulting effects on Aβ generation [21]. As mentioned above, Aβ is also known to increase both BACE1 and AβPP levels via DNA Aβ-interacting domains (AβID) in the AβPP and BACE1 promoters resulting in a feedback loop that increases Aβ production [51]. These multiple interactions reveal that the amyloidogenic pathway story in Alzheimer’s is far from complete and that CaM and Aβ lie at the heart of this critical stage in the disease.

## 5. Conclusions

**Figure 3:**
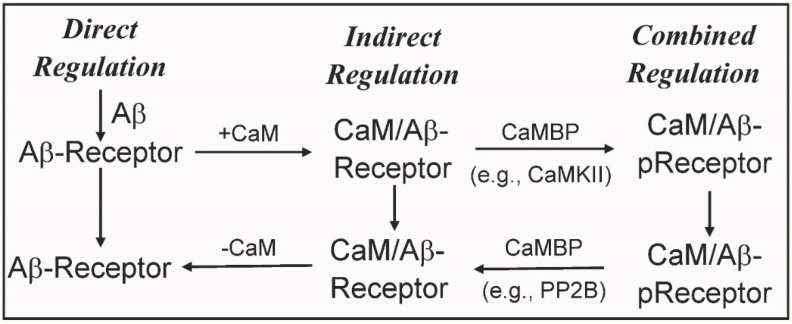
Some types of regulation open to Aβ receptors that are calmodulin binding proteins. pReceptor = phosphorylated receptor. See text for details.

The existence of Aβ receptors that are CaMBPs or are regulated by CaMBPs has revealed new levels of regulation that are only beginning to be understood. As evidenced above Aβ receptors can show direct regulation or indirect regulation. Research detailed above also provided insights into the complexity of combined regulation. These events are summarized in Figure 3 with CaMKII used as an example for events involved in combined regulation. Examples for each of these regulatory events were detailed above. The figure also reveals another series of potential regulatory options with reversions from one type of regulation (e.g., combined regulation) to another (e.g., indirect regulation). As a simple example, Indirect Regulation could be reversed by the removal of CaM. The impact of these three Aβ receptor CaM-based regulatory mechanisms on normal cell function and in neurodegenerative diseases requires further analysis.

This complex interplay between CaM, CaMBPs and Aβ-receptors may explain why no successful therapy has been developed to treat the various forms of AD. For example, attempts to treat AD by inhibiting BACE1 have not only been unsuccessful, but they have also led to confusing and, sometimes, contradictory results [1,52]. This could be explained both by the multiple normal physiological functions of Aβ in cells and/or by the multifaceted interplay between this CaM-binding peptide, its CaMBP/Aβ-receptors and the concomitant regulatory role of CaM and other CaMBPs, such as CaMKII and PP2B, on those receptors. With the multitude of critical CaM-binding and Aβ-binding proteins involved in the onset and progression of AD, many of which are the same, it seems prudent to continue this area of research. Determining the concentrations and intracellular locations of CaM, Aβ and the relevant CaMBPs in brain regions in normal and AD at selected stages (e.g., preclinical, MCI, dementia) versus non-AD brain regions could provide more insight into the impact of each of these components and their potential level of interplay.

## Figures and Tables

**Table 1:** Calmodulin Regulation of Aβ Receptors linked to Alzheimer’s Disease.

A. DIRECT REGULATION			
**1. Validated CaMBPs**			
***Aβ Receptor***	***Example Function***	***CaMBP Reference***	***Aβ Receptor Reference***
Aβ	oligos/fibrils/plaques	[11]	Not applicable
AβPP1	source of Aβ	[12]	[13]
mGluR	Ca^2+^ homeostasis	[14]	[15]
NMDAR	Ca^2+^ homeostasis	[16]	[17]
PMCA	Ca^2+^ homeostasis	[18]	[19]
PSEN-1	γ-secretase subunit	[20]	[21]
**2. Presumptive CaMBPs**			
***Aβ Receptor***	***Example Function***	***CaMBP Reference***	***Aβ Receptor Reference***
APOE 2-4	risk factor	[9]	[22]
CLU/ApoJ	risk factor	[9]	[23]
PICALM	risk factor	[9]	[24]
TREM2	risk factor	[10]	[25]
**B. INDIRECT REGULATION**			
***Aβ Receptor***	***Example Function***	***CaMBP Reference***	***Aβ Receptor Reference***
α7nAChR	Ca^2+^ homeostasis	Regulated by CaMKII	[26]
AMPAR	Ca^2+^ homeostasis	Regulated by PP2B	[27]
β2AR	adrenergic function	Regulated by CaMKII	[28]

Legend. α7nAChR, a7 nicotinic acetylcholine receptor; Aβ, amyloid β; AβPP1, amyloid β precursor protein 1; AdoA2, adenosine receptor A2; AMPAR, α-Amino-3-hydroxy-5-methyl-4-isoxazolepropionic acid receptor; APOE 2-4, apolipoprotein E 2-4; β2AR, β2 adrenergic receptor; CaMKII, calcium/calmodulin dependent protein kinase II; Ca_v_2, L-type Ca Channel; CLU/ApoJ, clusterin/apolipoprotein J; D2DR, D2 Dopamine Receptor; mAchR, metabotropic muscarinic receptor; mAchR, metabotropic glutamine receptor; NMDAR, N-methyl-D-aspartate receptor; PICALM, Phosphatidylinositol-binding clathrin assembly protein; PP2B, protein phosphatase 2b, calcineurin; PSEN-1, presenilin-1; TREM2, triggering receptor expressed on myeloid cells 2.

## Data Availability

Not applicable.

## Abbreviations

Aβ	amyloid beta
Aβo	amyloid beta oligomers
AβPP	amyloid-β precursor protein
AchR	acetylcholine receptor
AD	Alzheimer’s disease
APOE	apolipoprotein E
AMPAR	α-amino-3-hydroxy-5-methyl-4-isoxazolepropionic acid receptor
BACE1	beta-secretase 1
BIN1	bridging Integrator 1
CaM	calmodulin
CaMBD	calmodulin binding domain
CaMBP	calmodulin binding protein
CaMKII	calcium/CaM-dependent kinase II
PP2B	calcineurin
CLU	clusterin
CRAC	calcium release-activated calcium channels
CR1	complement receptor type 1
LTP	long-term potentiation
LTD	long-term depression
MARCKs	myristoylated alanine-rich, C-kinase substrate
mGluR5	metabotropic glutamate receptor 5
NFTs	neurofibrillary tangles
Ng	neurogranin
NMDAR	N-methyl-D-aspartate receptor
PMCA	plasma membrane calcium ATPase
pTau	phosphorylated Tau
RyRs	ryanodine receptors
TREM2	triggering receptor expressed on myeloid cells 2
